# Calcium Chloride in Neonatal Parenteral Nutrition: Compatibility Studies Using Laser Methodology

**DOI:** 10.1371/journal.pone.0106825

**Published:** 2014-09-05

**Authors:** Robert K. Huston, J. Mark Christensen, Chanida Karnpracha, Jill E. Rosa, Sara M. Clark, Evelyn A. Migaki, YingXing Wu

**Affiliations:** 1 Northwest Newborn Specialists, PC and Pediatrix Medical Group, Portland, OR, United States of America; 2 Department of Pharmaceutical Sciences, College of Pharmacy, Oregon State University, Corvallis, OR, United States of America; 3 Pharmacy Department, Denver Veterans Affairs Medical Center, Denver CO, United States of America; 4 Neonatal Pharmacy, Providence St. Vincent Medical Center, Portland, OR, United States of America; 5 Medical Data Research Center, Providence Health and Services, Portland, OR, United States of America; University of Missouri-Kansas City, United States of America

## Abstract

**Introduction:**

We have previously reported results of precipitation studies for neonatal parenteral nutrition solutions containing calcium chloride and sodium phosphate using visual methods to determine compatibility. The purpose of this study was to do further testing of compatibility for solutions containing calcium chloride using more sensitive methods.

**Methods:**

Solutions of Trophamine (Braun Medical Inc, Irvine, CA) and Premasol (Baxter Pharmaceuticals, Deerfield, IL) were compounded with calcium chloride and potassium phosphate. Controls contained no calcium or phosphate. After incubation at 37° for 24 hours solutions without visual precipitation were analyzed to determine mean particle size using dynamic light scattering from a laser light source.

**Results:**

Particle sizes were similar for control solutions and those without visual precipitation and a mean particle size <1000 nm. Compatible solutions were defined as those with added calcium and phosphate with no visual evidence of precipitation and mean particle size <1000 nm. In solutions containing 2.5–3% amino acids and 10 mmol/L of calcium chloride the maximum amount of potassium phosphate that was compatible was 7.5 mmol/L.

**Conclusion:**

Maximum amounts of phosphate that could be added to parenteral nutrition solutions containing Trophamine and calcium chloride were about 7.5–10 mmol/L less for a given concentration of calcium based upon laser methodology compared to visual techniques to determine compatibility. There were minor differences in compatibility when adding calcium chloride and potassium phosphate to Premasol versus Trophamine.

## Introduction

Associations between aluminum (Al) exposure from contamination of parenteral solutions and impaired neurological development as well as reduced bone mass have previously been reported [1]–[2]. Aluminum is a contaminant introduced into many small and large volume parenteral products during the manufacturing process, as well as due to leaching of Al during sterilization of glass containers [3]. Calcium gluconate (CaGlu) is commonly used in parenteral nutrition (PN) solutions due to decreased dissociation of calcium cations into solution, thereby decreasing the risk of forming precipitates with phosphate, compared to calcium chloride (CaCl_2_) [4]. At the concentrations of calcium and phosphorus used in PN solutions to support neonatal patients the risk of precipitation is increased [5]. However, CaGlu has been consistently identified as the calcium (Ca) additive with the highest level of Al contamination in PN [6]–[8] while PN solutions made with CaCl_2_ and sodium phosphate (NaPhos) contain significantly less Al compared to those made with CaGlu and potassium phosphate (KPhos) [1], [9]. Currently, the FDA recommends limiting the Al intake from contamination of PN for preterm infants to ≤0.19 µmol/kg/day (≤5 µg/kg/day) [10]. Poole et al have shown that it is not possible to meet this goal and provide acceptable calcium and phosphorus intakes using neonatal PN solutions containing CaGlu and KPhos [11]. Calcium chloride is the only calcium additive available in North America that, when added to neonatal PN solutions, limits inadvertent administration of Al to levels near the FDA recommendation.

Previously published results of precipitation studies for CaCl_2_ and NaPhos in TrophAmine from our neonatal pharmacy utilized visual methods for detecting precipitation [9] Letters to the editor subsequent to publication of those results discussed the need for more sensitive techniques for determining compatibility of Ca and phosphate (Phos) in PN solutions in order to establish safe guidelines for use of solutions containing CaCl_2_ and Phos in neonatal PN [12]–[13]. Therefore, further studies were conducted on neonatal PN solutions containing CaCl_2_ using laser and microscopic techniques to detect evidence of precipitation in PN solutions compounded with two of the available neonatal amino acid (AA) additives. The results of these studies are presented in this paper.

## Methods

This study was conducted from February 27 through May 30, 2013. All study solutions were compounded by a neonatal pharmacist in the Neonatal Intensive Care Unit (NICU) pharmacy at Providence St. Vincent Medical Center in clear plastic Exacta Mix 250 mL EVA containers (Baxa Corporation, Englewood, CA) using a Baxa Exactamix 2400 Compounder (Baxa Corporation, Englewood, CO). Additives that were added to all solutions are shown in Table 1. Total final volume of each solution was 100 mL. Study solutions containing TrophAmine (Braun Medical Inc., Irvine, CA) had final concentrations of 1%, 1.5%, 2%, 2.5%, 2.8%, 3%, 3.5%, and 4% AA. Calcium chloride (Hospira Inc, Lake Forest, IL or International Medication Systems, South El Monte, CA) was added in concentrations of 2.5, 5, 7.5, 10, 12.5, and 15 mmol/L while KPhos (Hospira Inc, Lake Forest, IL) was added in concentrations of 5, 7.5, 10, 12.5, 15, and 20 mmol/L. Solutions containing Premasol (Baxter Pharmaceuticals, Deerfield, IL) were examined in concentrations of 1.5%, 2%, 2.5%, 2.8%, 3%, and 3.5% AA with CaCl_2_ of 2.5, 5, 7.5, 10, and 12.5 mmol/L and KPhos of 5, 7.5, 10, 12.5, and 15 mmol/L. For both Trophamine and Premasol, AA concentrations were studied in order from lowest to highest concentration. Based upon results for lower AA concentrations not all concentrations of CaCl_2_ and KPhos were studied in each AA concentration. For example, if solutions containing the same concentrations of Ca and Phos were compatible in AA concentrations of 1.5% and 2% AA we did not necessarily continue to test the compatibility of these concentrations of Ca and Phos in 2.5% and 3% AA. Likewise, based upon data from our previous experience [9], if it was unlikely that high concentrations of Ca and Phos would be compatible in low AA concentrations those combinations of additives were not studied. Fourteen controls (one for each AA concentration of Trophamine and Premasol) without any added CaCl_2_ or KPhos were also compounded.

**Table 1:** Standard Parenteral Nutrition Additives for All Solutions Compounded in the Precipitation Studies.

Additive	Manufacturer	Total Dose
Dextrose 70%	Hospira, Inc., Lake Forest, IL	10 g
Sterile Water	Hospira, Inc., Lake Forest, IL	QS to 100 mL
Heparin (100 units/mL)	APP Pharmaceuticals, Schaumburg, IL	50 units
Magnesium Sulfate 50% (4 mEq/mL)	APP Pharmaceuticals, Schaumburg, IL	0.5 mEq
Zinc Choride (1 mg/mL0	Hospira, Inc., Lake Forest, IL	400 µg
Copper Sulfate (400 mcg/mL)	American Regent, Inc., Shirley, NY	20 µg
Selenium (40 µg/mL)	American Regent, Inc., Shirley, NY	2 µg
Levocarnitine (200 mg/mL)	Sigma-Tau Pharmaceuticals, Gaithersburg, MD	5 mg
Sodium Acetate (2 mEq/mL)	Hospira, Inc., Lake Forest, IL	2 mEq
Potassium Chloride (2 mEq/mL)	Hospira, Inc., Lake Forest, IL	1 mEq

After compounding, solutions were transported in the PN bag by automobile (time for transport: 1.5 hr) to the Department of Pharmaceutical Sciences at Oregon State University where they were incubated at 37°C for 24 hours in a warming oven. Bags were visualized in a dark room while trans-illuminating the solution with a bright beam of light before and after incubation to determine evidence of precipitation. Solution pH was measured on a 3 mL sample before and after incubation using a pH meter (Mettler Toledo FIVE™, Schwerzenbach, Switzerland). After incubation three 1 mL samples from each bag were then analyzed using a laser instrument (Zetasizer Nano ZS, Model ZEN3600, Malvern Instruments Ltd, Worcestershire, UK) to determine the average particle size in solutions that did not show evidence of visual precipitation in the dark room. The Zetasizer uses dynamic light scattering to measure the hydrodynamic diameter of the particle and computes the average particle size for the range of particles in a given solution (the Z average particle size). It also computes the polydispersity index (PDI) for the Z average particle size distribution curve. The PDI is equal to the square of the standard deviation (SD) of the width of the curve (in nm) divided by the square of the average particle size for the particle size distribution. With the software used in this study, diameters of 0.3–50,000 nm are able to be detected by this instrument when computing the Z average particle size. The Zetasizer can also determine sub-peaks for particles in the range of 0.3–10,000 nm if the PDI for the sub-peak curve is less than 0.08 (SD of the width of the curve <30% of the average particle size for the sub-peak). All particle size distributions were reviewed by one of the authors (JMC) in order to identify any sub-peaks having an average particle size that might exceed the smallest capillary size of 4000 nm. Samples (0.5 mL) were then analyzed with a NanoSight microscope (NanoSight Ltd, Wiltshire, UK) which uses a laser light source and computerized video recording software to determine the average particle size and particle count for each solution. Optimum particle sizes detected by this instrument are 10–2000 nm. All gross visualization of solutions in the dark room, as well as the Zetasizer and microscopic measurements were performed by the same pharmaceutical sciences graduate student.

In order to determine the predicted particle size at the extreme high end of the particle size distribution curve, the computed value was determined using the Zetasizer Z average diameter plus 3 SD above the particle size mean for each solution. The Z average diameter of the particle size distribution determined using the Zetasizer (average of the 3 measurements) and the Z average plus 3 SD, the average particle size as well as the particle counts determined by the microscope, and pH measurements for each solution were entered into an Excel spreadsheet (Microsoft Corp., Redmond, WA). Means, standard deviations, medians, and range for each measurement were determined using the Excel Spreadsheet software. Student's *t* tests for normally distributed continuous data and the Wilcoxon rank sum test for non-normally distributed continuous data were performed by a statistician to evaluate differences in particle size and particle counts between controls with no added Ca and Phos and solutions grouped as follows: those with no evidence of visual precipitation and a Z average particle size <1000 nm as determined by the Zetasizer (Group 1), those with no evidence of visual precipitation and a Z average particle size >1000 nm (Group 2). The value of 1000 nm was chosen because solutions with a Z average particle size <1000 nm were within the range found for the control solutions. Measurements of pH of solutions of Trophamine compared to solutions of Premasol were also compared.

The study was approved by the Institutional Review Board for Providence Health and Services, Portland, Oregon. No formal review was required since no human subjects were involved in the study.

## Results

A total of 271 solutions were compounded and 128 solutions precipitated visually in the dark room. Data for mean particle size as measured by the Zetasizer and the microscope are shown in Table 2 for solutions that did not precipitate visually in the dark room. The mean particle size as measured by the microscope for both study groups was lower than the particle size for controls but particle counts were not significantly different. The microscope has a narrow range for particle size detection as noted above, however. Solutions in Group 1 had a mean particle size as determined by the Zetasizer similar to controls without added Ca and Phos. The median (range) for the Z average plus 3 SD above the mean for controls was 601 (151–3745) nm compared to 622 (82–3603) nm for Group 1. None of these solutions had sub-peaks with a mean particle size greater than 4000 nm in diameter. Most solutions were characterized by having a single peak, less often two peaks, and least often three peaks.

**Table 2:** Average Particle Size for Solutions without Precipitation.

Group	N	Zetasizer (nm)	PDI	Micro (nm)	Micro(PC^a^)
C (Mn ± SD)	14	301±326	0.489±0.175	276±94	205±157
C (Md [R])	14	203 [44–1204]	0.489 [0.179–0.731]	261 [151–451]	167 [19–475]
G1 (Mn ± SD)	90	330±261	0.450±0.175	223±99^b^	188±130
G1 (Md [R])	90	223 [29–996]	0.400 [0.153–0.831]	193 [132–674]	182 [17–901]
G2 (Mn ± SD)	39	3241±3255^b^	0.885±0.118^c^	217±87^d^	145±101
G2 (Md [R])	39	1812 [1026–14563]	0.921 [0.636–1.000]	196 [107–564]	120 [33–539]

C, control; G1, group 1; G2, group 2; Micro, microscopic; Mn, mean; Md, median; PC, particle count; PDI, polydispersive index; R, range; SD, standard deviation.

a x10^6^/mL.

b p<0.01 compared to Control, Wilcoxon rank sum test.

c p<0.01 compared to Control, Student's *t* test.

d p<0.02 compared to Control, Wilcoxon rank sum test.

Based upon these results, compatible solutions were defined as those that had a Z average particle size as determined by the Zetasizer of <1000 nm and showed no visual evidence of precipitation. Maximum amounts of elemental Ca (as CaCl_2_) that could be added to solutions of Trophamine with varying amounts of KPhos that met the above criteria for compatibility are shown in Table 3. Maximum amounts of elemental Ca (as CaCl_2_) that could be added to solutions of Premasol with varying amounts of KPhos are shown in Table 4. There were minimal differences in the amount of Phos that could be added for a given concentration of CaCl_2_ between Trophamine and Premasol. For both Trophamine and Premasol the maximum amount of Phos that was compatible with 10 mmol/L of CaCl_2_ (a minimum concentration of Ca that may allow adequate intakes in neonatal PN) was 7.5 mmol/L in solutions that contained an AA concentration of greater than or equal to 2.5%.

**Table 3:** Maximum Concentrations (mmol/L)^a^ of Elemental Calcium (as CaCl_2_) Allowable Without Precipitation at Various Trophamine and Potassium Phosphate Concentrations.

Amino Acid	KPhos	KPhos	KPhos	KPhos	KPhos
g/L (%)	5 mmol/L	7.5 mmol/L	10 mmol/L	12.5 mmol/L	15 mmol/L
10 (1%)	2.5	2.5	2.5	0	0
15 (1.5%)	12.5	2.5	2.5	0	0
20 (2%)	12.5	7.5	5	5	0
25 (2.5%)	15	10	5	5	0
28 (2.8%)	15	10	7.5	5	2.5
30 (3%)	15	10	7.5	5	2.5
35 (3.5%)	15	10	10	5	5
40 (4%)	15	10	10	7.5	7.5

CaCl_2_, calcium chloride; KPhos, potassium phosphate.

a One mmol of calcium equals 40 mg or 2 mEq.

**Table 4:** Maximum Concentrations (mmol/L)^a^ of Elemental Calcium (as CaCl_2_) Allowable Without Precipitation at Various Premasol and Potassium Phosphate Concentrations.

Amino Acid	KPhos	KPhos	KPhos	KPhos	KPhos
g/L (%)	5 mmol/L	7.5 mmol/L	10 mmol/L	12.5 mmol/L	15 mmol/L
15 (1.5%)	12.5	5	5	0	0
20 (2%)	12.5	7.5	5	0	0
25 (2.5%)	12.5	10	5	2.5	2.5
28 (2.8%)	12.5	10	5	2.5	2.5
30 (3%)	12.5	10	5	2.5	2.5
35 (3.5%)	12.5	12.5	7.5	7.5	2.5

CaCl_2_, calcium chloride; KPhos, potassium phosphate.

a One mmol of calcium equals 40 mg or 2 mEq.

Table 5 displays average pH values for control solutions with AA concentrations of 1.5–3.5% with no added Ca or Phos prior to incubation (0 hours) and after incubation for 24 hours for both Trophamine and Premasol. Also displayed are the average pH values for all solutions containing any Ca with KPhos concentrations of 7.5 and 15 mmol/L at time 0 and those with the same concentrations of KPhos that did not precipitate compared to those that did precipitate after incubation for 24 hours. No significant differences were observed in pH relative to Ca content so values for solutions containing from 2.5–15 mmol/L of Ca were averaged. In contrast, adding Phos increased pH and, while there was little change in pH between 0 and 24 hours of incubation at 37°C for solutions that did not precipitate, there was a decrease in pH for Trophamine solutions that did precipitate. Although the pH of controls was lower for solutions with Premasol compared to Trophamine, the pH of the solutions after adding Ca and KPhos was similar after incubation for 24 hours for solutions that did not precipitate but lower for Trophamine compared to Premasol for solutions that did precipitate.

**Table 5:** pH (Mean ± SD) for Control Solutions with No Added Calcium or Phosphate and Those with Any Added Calcium and Potassium Phosphate (mmol/L).

Incubation	0 hours	24 hours	0 hours	24 hours	24 hours	0 hours	24 hours	24 hours
	Control	Control		No PPt	PPT		No PPt	PPT
KPhos	0	0	7.5	7.5	7.5	15	15	15
Trophamine	5.81±0.04	5.77±0.05	5.95±0.07	5.90±0.05	5.67±0.25	6.06±0.09	6.02±0.09	5.57±0.14
N	6	6	20	15	5	32	14	19
Premasol	5.61±0.05^a^	5.63±0.04^a^	5.80±0.07^a^	5.82±0.09^b^	5.83±0.09	5.93±0.08^a^	5.94±0.10	5.81±0.16^a^
N	6	6	11	7	4	19	6	13

KPhos, potassium phosphate; PPt, precipitate.

a p<0.001 compared to Trophamine, Student's *t* test.

b p<0.02 compared to Trophamine, Student's *t* test.

## Discussion

This study is the first to report Ca and Phos precipitation studies for PN solutions containing Premasol for a range of concentrations of Ca, Phos, and AA. According to the manufacturers, the pH range and the individual AA concentrations of Premasol are almost identical to those for Trophamine, which would suggest that results of precipitation studies for Ca and Phos for the two AA additives should be similar. The findings presented here appear to support this assumption. Although there were some minor differences in the amount of Ca that could be added based upon our criteria for compatibility, these may be related to AA concentration differences for the lots of the solutions that were studied or due to variation related to compounding solutions for each AA product concentration on different days. Although the pH of the Premasol solutions was lower initially, adding Phos increased the pH of Premasol solutions to values similar to those for Trophamine, which would suggest that pH differences between the two AA additives did not significantly affect precipitation. It is unclear why the pH of the solutions that precipitated was lower for solutions containing Trophamine compared to Premasol.

As would be expected, the amount of Ca and Phos that could be added to PN solutions containing CaCl_2_ as the Ca additive was less in this study using more sensitive methods to determine compatibility than that reported in a previous study using visual methods only [9]. One difference between the studies is that NaPhos was used as the Phos additive in the previous study due to its lower Al content (approximately 8–9 µ/kg/day less Al) [9] versus KPhos in the current study because of the national shortage of NaPhos. It is possible that there may be differences in precipitation rates for solutions containing NaPhos versus KPhos. For AA solutions with an AA concentration of ≥2.5% the difference appeared to be 7.5–10 mmol/L less Phos that could be added for a given concentration of Ca. The only other study to use laser technology to determine Ca and Phos compatibility for a range of concentrations of AA, Ca, and Phos in Trophamine studied solutions containing CaGlu and KPhos with added cysteine [14]. Compared to an earlier study using visual methods [15] for a solutions of Trophamine with the same constituents, 5–15 mmol/L less Phos appear to be able to be added based upon laser studies of compatibility versus studies using visual methods. Thus, the current study of CaCl_2_ appears to be consistent with studies of CaGlu when comparing visual to laser methodology.

A possible limitation of the current study may relate to the use of the Zetasizer to evaluate particle size. This instrument, which uses dynamic light scattering (DLS) rather than light obscuration (LO) when subjecting samples of solution to a laser beam, is not able to accurately perform particle counts [16]. Although counts of particles of sizes greater than 10,000 nm (10 microns) using LO have been employed by some investigators to determine compatibility of Ca and Phos in PN solutions, the criteria for determining compatibility using this method have not been consistent [17]–[20]. Chaieb et al [21] used a dynamic light scattering instrument (Nanosizer ZS, Malvern Instruments Ltd, Worcestershire, UK) to determine mean particle size and define compatibility but found that the usefulness of these measurements were limited due to the narrower range of values for particle size detectable by this instrument (0.6–6000 nm) compared to the Zetasizer. The study of Hoie and Narducci [14] used laser diffraction (LD) and employed a semi-quantitative method that relied predominantly on an estimation of solution density. It is the only study mentioned in the current Handbook of Injectable Drugs as a reference for compatibility for a full range of AA, Ca, and KPhos concentrations in Trophamine that used laser methods, while references to other compatibility studies for full ranges of added AA, Ca, and Phos in Trophamine employed visual and microscopic methods [22].

At this time more studies are needed to determine the optimal methodology when evaluating the compatibility of Ca and Phos in PN solutions. Studies of PN solutions containing lipid emulsions (3 in 1 PN solutions) have indicated that LO is more accurate than LD when determining the fraction of particles >5000 nm in the high end tail of the lipid emulsion particle size distribution curve [23]. Two of three instruments that employed LD overestimated the fraction of particles >5000 nm. Studies demonstrate that DLS is very accurate when evaluating particle sizes <1300 nm compared to LO whereas LO is more accurate at larger diameters [24]. Thus far there are no studies that have shown that one method (LO, LD, or DLS) is more sensitive than the others in order to establish criteria for compatibility of Ca and Phos in PN solutions.

While most references indicate that 4000 nm is the diameter of the smallest capillaries there are no in vivo studies that we are aware of to verify this value in preterm infants. An in vitro study using tissue culture of embryos and fetuses of from approximately 15–21 weeks gestation found the smallest capillaries to be 2000–8000 nm [25]. It was noted that in vitro techniques may lead to underestimation of capillary diameter compared to in vivo studies, however. The above noted studies using LO to determine incompatibility by measuring particle size counts >10,000 and >25,000 nm as recommended by USP (United States Pharmacopeia) 788 [18] do not address particle sizes in the range of the smallest capillaries. It may be that a combination of methods is needed in order to determine the compatibility of Ca and Phos in neonatal PN, such as DLS to assure that the PN solution particle size profile for particles less than 2000 nm are indeed less than 2000 nm, as well as, LO or other methods to assure that particle counts for larger particles meet appropriate criteria.

A second possible limitation of this study is that we used a single dextrose concentration of 10% for all solutions. Previous studies, however, have not demonstrated any significant affect of dextrose concentration on Ca and Phos compatibility in PN solutions when comparing 10% versus 25% dextrose [26] or when comparing 5% versus 10% dextrose [19], [20].

Results of this study suggest that the maximum amount of KPhos that can be added safely to solutions with AA concentrations of ≥2.5% (AA concentrations most often found in neonatal PN solutions) and containing at least 10 mmol/L of CaCl_2_ is 7.5 mmol/L compared to 15 mmol/L of NaPhos in a previous study [9]. Using concentrations that appeared to be safe in the previous study, the amounts of Ca and Phos that could be administered parenterally using CaCl_2_ as the preferred additive to neonatal PN containing Trophamine were able to meet recommended guidelines for parenteral intakes of both Ca and Phos of 1.5–2 mmol/kg/day for preterm infants [27] and minimize Al exposure [28]. It does not appear to be possible to provide these intakes of Ca and Phos when CaCl_2_ is the Ca additive to PN while limiting concentrations of Ca and Phos to amounts determined to be compatible using laser methodology as presented in this study. On the other hand, using solutions containing CaGlu and cysteine in Trophamine, the only other solution for which there is a laser based curve for compatibility available, increases Al intake to levels found to adversely affect bone mineralization and neuro-developmental outcome [1], [9], [11], [29].

In conclusion, this study presents guidelines for limits for Ca and Phos concentrations that can be used when compounding neonatal PN solutions containing Trophamine or Premasol without added cysteine when CaCl_2_ is the Ca additive. In this time of ongoing PN additive shortages these guidelines may be helpful when considering available alternatives. The results presented here found that, while CaCl_2_ is the preferred additive that is available in North America when attempting to reduce Al intake to levels near recommended limits, Ca and Phos administration will often be below recommended levels due to the need to limit the concentration of Ca and Phos in PN solutions containing CaCl_2_ and phosphates. There is no Ca additive available in North America that meets the requirement to limit Al exposure to near recommended levels and also provides optimal Ca and Phos administration for preterm infants. The results of this study emphasize the need to find a better alternative for adding Ca to neonatal PN in North America. CaGlu in plastic vials, as opposed to glass vials, has been available in Europe, but not North America, and is as low in Al content as CaCl_2_ [30]. Alternatively, if an organic phosphate preparation that is low in Al content and compatible with CaCl_2_ at higher concentrations of Ca and Phos could be made available in North America, CaCl_2_ could be used as the preferred Ca additive.

## Supporting Information

Data S1 (XLS)
